# The mitochondrial genome of *Angiostrongylus mackerrasae* as a basis for molecular, epidemiological and population genetic studies

**DOI:** 10.1186/s13071-015-1082-0

**Published:** 2015-09-17

**Authors:** Mahdis Aghazadeh, Rebecca J. Traub, Namitha Mohandas, Kieran V. Aland, Simon A. Reid, James S. McCarthy, Malcolm K. Jones

**Affiliations:** School of Veterinary Science, University of Queensland, Queensland, 4343 Australia; QIMR Berghofer Medical Research Institute, Brisbane, Queensland 4006 Australia; Faculty of Veterinary and Agricultural Sciences, The University of Melbourne, Victoria, 3052 Australia; Queensland Museum and Sciencentre, Queensland, 4101 Australia; School of Public Health, University of Queensland, Queensland, 4006 Australia

**Keywords:** *Angiostrongylus mackerrasae*, Mt genome, Illumina sequencing, Rat lungworm, Metastrongyloidea

## Abstract

**Background:**

*Angiostrongylus mackerrasae* is a metastrongyloid nematode endemic to Australia, where it infects the native bush rat, *Rattus fuscipes*. This lungworm has an identical life cycle to that of *Angiostrongylus cantonensis*, a leading cause of eosinophilic meningitis in humans. The ability of *A. mackerrasae* to infect non-rodent hosts, specifically the black flying fox, raises concerns as to its zoonotic potential. To date, data on the taxonomy, epidemiology and population genetics of *A. mackerrasae* are unknown. Here, we describe the mitochondrial (mt) genome of *A. mackerrasae* with the aim of starting to address these knowledge gaps.

**Methods:**

The complete mitochondrial (mt) genome of *A. mackerrasae* was amplified from a single morphologically identified adult worm, by long-PCR in two overlapping amplicons (8 kb and 10 kb). The amplicons were sequenced using the MiSeq Illumina platform and annotated using an in-house pipeline. Amino acid sequences inferred from individual protein coding genes of the mt genomes were concatenated and then subjected to phylogenetic analysis using Bayesian inference.

**Results:**

The mt genome of *A. mackerrasae* is 13,640 bp in size and contains 12 protein coding genes (*cox*1-3*, nad*1-6*, nad*4L*, atp*6 and *cob),* and two ribosomal RNA (rRNA) and 22 transfer RNA (tRNA) genes.

**Conclusions:**

The mt genome of *A. mackerrasae* has similar characteristics to those of other *Angiostrongylus* species. Sequence comparisons reveal that *A. mackerrasae* is closely related to *A. cantonensis* and the two sibling species may have recently diverged compared with all other species in the genus with a highly specific host selection. This mt genome will provide a source of genetic markers for explorations of the epidemiology, biology and population genetics of *A. mackerrasae*.

## Background

The rat lungworm, *Angiostrongylus cantonensis,* the cause of neural angiostrongyliasis in humans and animals has been described from most inhabited continents, including Australia. Another two of the 19 species of this genus are neurotropic, namely *A. malaysiensis*, a parasite of the forest rat, *Rattus tiomanicus* [1–3] in Southeast Asia, and *A. mackerrasae,* a parasite of the native bush rats, *Rattus fuscipes* and *R. Leucopus* of Australia*.* The latter species of *Angiostrongylus* appears to occur in sympatry with *A. cantonensis* in Australia. To date, the genetic identity of the Australian native species of the rat lungworm*, A. mackerrasae,* has not been explored and there is no sequence data available for this species. Despite small morphological differences between the two species of *Angiostrongylus* present in Australia [4], it is uncertain if the morphological differences are accompanied by sufficient genetic divergence so as to support the concept that the two are indeed distinct species. Although *A. mackerrasae* is not known to infect humans, the ability of the parasite to produce patent infections in the lungs of the black flying fox (*Pteropus alecto*) [5], also raises questions as to the pathogenicity of this species in non-permissive hosts, including humans.

*Angiostrongylus mackerrasae* has been distinguished from the sympatric *A. cantonensis,* on the basis of distinct morphology of the reproductive system. For adult male *A. cantonensis,* the average length of the copulatory spicules is 1.24 mm, compared with 0.49 mm for *A. mackerrasae* [4]. A morphometric analysis of 51 adult females of *A. cantonensis* and 64 adult females of *A. mackerrasae* by Bhaibulaya [4] revealed that the mean length of the vagina of *A. cantonensis* was 2.10 mm, whereas for *A. mackerrasae* it was 1.39 mm. However, there is an overlap in the range of vaginal length between the two species, making it difficult to identify the species by examining only the adult female [6]. Additionally, the adult female of *A. mackerrasae* possesses a minute terminal projection at the tip of the tail, which in *A. cantonensis* is absent. Despite these morphological (phenetic) differences, little information is available on the epidemiology of these sympatric species in Australia. Much remains to be investigated, including their host range, whether mixed species infections in the definitive hosts occur and, if so, whether they are capable of producing hybrids in nature.

An important advance in understanding the epidemiology of *Angiostrongylus* species in Australia would arise from better understanding on genetic divergence of *A. mackerrasae* and *A. cantonensis.* Genetic markers, together with morphological characters, could be used to identify parasites associated with disease in humans, domestic and wild animals, as well as investigate the geographical distribution and host selection of *A. mackerrasae* and *A. cantonensis* in the large diversity of *Rattus* species that occur in Australia [7] and their intermediate hosts [8], areas hitherto unexplored. In the present study, we took a first step towards addressing some of these areas by characterising the mt genome of *A. mackerrasae* as a rich source of genetic markers. We also genetically compared, for the first time, *A. mackerrasae* with its very closely related congener, *A. cantonensis*.

## Methods

### Sample collection and DNA extraction

#### Ethical approval

All animal experiments were approved by the Animal Ethics Committee of the QIMR Berghofer Medical Research Institute (project P1457) and ratified by the University of Queensland Animal Welfare Unit. Specimens of *Rattus fuscipes* were collected from the Department of Environment and Heritage Protection of the Queensland Government (permit WIS12109412). Specimens of *Rattus fuscipes* were trapped in Brisbane and surrounding regions using Eliot traps, baited with peanut butter and rolled oats. Rat faeces were directly examined by light microscopy for the presence of larvae consistent with *Angiostrongylus* sp. [3]; rats harbouring the parasite were euthanized with an overdose of CO_2_ in a portable chamber for subsequent transport to the laboratory. Specimens of *Angiostrongylus* recovered from the pulmonary arteries of infected rats were identified to species morphologically [4] and washed extensively in physiological saline. Genomic DNA was isolated from amid-body section (to avoid ovaries) of an individual adult female worm using the QIAGEN DNeasy blood and tissue extraction kit, according to manufacturer’s instructions (Qiagen, Germany).

#### Long PCR amplification

The complete mt genome of a single *A. mackerrasae* female worm was amplified by long-PCR using a high fidelity PCR enzyme (BD Advantage 2, BD Biosciences) as two overlapping amplicons (~8 kb and 10 kb) as described [9], using modified primers (Table 1) and an optimised annealing temperature (58 °C), employing a suitable positive (*A. cantonensis* DNA recovered from Australian *Rattus rattus*) and negative (i.e. no template) controls. Individual PCR products were resolved in separate lanes on an agarose gel (1 % w/v) in TBE buffer (Tris/Borate/EDTA) and stained with SYBR®Safe gel stain (Life Technologies). Individual PCR products (~8 kb and 10 kb) were excised from the gel and purified using the QIAquick gel extraction kit (QIAGEN).

**Table 1:** Oligonucleotides used in this study

Oligonucleotide	Sequence	Position	Reference
5 F-Mod	TATATGAGCGTCATTTATTAGG	nad1	This study
44R-Mod	CTACCTTAATGTCCTCACGC	rrnL	This study
39 F	TCTTAGCGTGAGGACATTAAG	rrnL	Hu et al., 2007 [8]
42R-Mod	CCTAATAAATGACGCTCATAAG	nad1	This Study

#### Sequencing and data analyses

Short-insert libraries (100 bp) were constructed from the purified products and then sequenced using Mi-seq technology (Illumina platform; Yourgene, Taiwan). FastQC (Babraham Bioinformatics: www.bioinfomatics.babraham.ac.uk) was utilised to assess the quality of sequence data and the paired-end reads were filtered using Trimmomatic (http://www.usadellab.org/cms). De novo assembly of the sequences was performed using SPAdes 3.0.0 Genome Assembler (http://bioinf.spbau.ru/en/spades). The program was run for all odd k-mer sizes between 21 and 125 (inclusive). The k-mer size providing the largest scaffold was selected for further analysis.

Following assembly, the mt genome of *A. mackerrasae* was annotated using a semi-automated bioinformatic pipeline [10]. Each protein coding mt gene was identified by local alignment comparison (performed in all six frames) using amino acid sequences from corresponding genes from mt genomes of *A. vasorum*, *A. cantonensis* and *A. costaricensis*; accession nos.NC_018602, GQ398121 and GQ398122, respectively [11, 12]. The large and small subunits (*rrn*L and *rrn*S) of mt ribosomal RNA genes were identified by local alignment, and all transfer RNA (tRNA) genes were predicted and annotated based on available data from selected nematode superfamilies, (the Metastrongyloidea, Trichostrongyloidea, Ancylostomatidea and Strongyloidea). Annotated sequence data were imported using the program SEQUIN (available *via*http://www.ncbi.nlm.nih.gov/Sequin/) for the final verification of the mt genome organisation and subsequent submission to the GenBank database. The amino acid sequences translated from individual genes of the mt genome of *A. mackerrasae* were then concatenated and aligned to sequences for 18 species for which mt genomic data sets were available using the program MUSCLE [13].

Phylogenetic analysis of amino acid sequence data was conducted by Bayesian inference (BI) using Monte Carlo Markov Chain analysis in the program MrBayes v.3.2.2 [14]. Bayesian analysis is more widely accepted and more accurate than the other methods due to the integration of Markov chain monte carlo algorithm. The optimal model of sequence evolution was assessed using a mixed amino acid substitution model, with four chains and 200,000 generations, sampling every 100th generation; the first 25 % of the generations sampled were removed from the analysis as burn-in. In addition, a sliding window analysis was performed on the aligned, complete mt genome sequences of the three *Angiostrongylus* species using the program DnaSP v.5 (http://www.ub.edu/dnasp/).

A sliding window of 300 bp (steps of 10 bp) was used to estimate nucleotide diversity (π) over the entire alignment; indels were excluded using DnaSP. Nucleotide diversity for the entire alignments was plotted against midpoint positions of each window, and gene boundaries were defined. Pairwise analyses were also performed using amino acid sequences predicted from protein coding genes of the four *Angiostrongylus* species to identify regions of different magnitudes of amino acid diversity.

## Results

### Characteristics of mt genome of *A. mackerrasae*

**Fig. 1:**
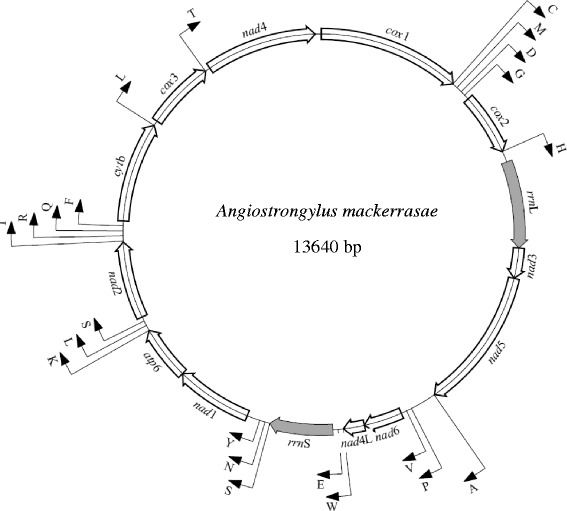
Schematic representation of the circular mitochondrial genome of *Angiostrongylus mackerrasae.* Each transfer RNA gene is identified by one letter amino acid code on the outer side of the map. All genes are transcribed in the clockwise direction

The circular mt genome of *A. mackerrasae* is 13,640 bp in length (Fig. 1), similar in length to those of *A. cantonensis* (13,497 bp), *A. costaricensis* (13,585 bp) [12] and *A.vasorum* (13,422 bp) [11]. Consistent with the pattern seen in other metastrongyloids [11, 12, 15], the mt genome of *A. mackerrasae* is AT-rich, with T being the most frequent and C being the least frequent nucleotides. The nucleotide composition of the mt DNA of *A. mackerrasae* was 24.42 % for A, 20.81 % for G, 6.35 % for C and 48.42 % for T (Table 2). The mt genome contains 12 protein coding genes (*cox*1-3*, nad*1-6*, nad*4L*, atp*6and *cob)*, as well as two ribosomal RNA (rRNA) and 22 transfer RNA (tRNA) genes. All of the 36 genes are transcribed in the same direction (5’ > 3’) (Fig. 1).

**Table 2:** Nucleotide composition (%) for the entire or regions of the mitochondrial genome of *Angiostrongylus mackerrasae, Angiostrongylus cantonensis, Angiostrongylus vasorum and Angiostrongylus costaricensis*

Species		Length (bp)	A	C	T	G	A + T
*Angiostrongylus mackerrasae*	Entire sequence	13640	24.42	6.35	48.42	20.81	72.84
	Protein genes	10341	21.79	6.44	49.92	21.85	71.71
	RNA genes	1659	32.07	6.93	43.40	17.60	75.47
*Angiostrongylus vasorum*	Entire sequence	13646	21.13	6.04	46.85	24.33	67.98
	Protein genes	10579	18.49	6.05	48.2	25.53	66.69
	RNA genes	1688	29.98	6.22	42.3	19.79	72.28
*Angiostrongylus cantonensis*	Entire sequence	13722	24.2	6.1	48.0	20.2	72.2
	Protein genes	10642	21.6	6.2	49.4	21.4	71.0
	RNA genes	1688	31.7	6.0	43.7	16.9	75.4
*Angiostrongylus costaricensis*	Entire sequence	13812	25.0	6.5	47.0	20.0	72.0
	Protein genes	10514	22.5	6.7	48.4	20.8	70.9
	RNA genes	1692	32.3	6.4	42.3	17.3	74.6

### Protein genes

The initiation and termination codons were predicted for protein-encoding genes of *A. mackerrasae* and were then compared with those of *A. cantonensis*, *A. costaricensis* and *A. vasorum* (Table 3). The most common start and stop codons for *A. mackerrasae* was TTG (for 6 of the 12 proteins) and TAG (for 6 of the 12 proteins). The codon usage of the 12 protein coding genes was compared with *A. cantonensis*, *A. costaricensis* and *A. vasorum* (Table 4). The most frequently used codon was TTT (Phe) and TTG (Leu), similar to those in mt genomes of *A. cantonensis*, *A. vasorum* and *A. costaricensis*. In addition, the least frequently used codons in the mt genome of *A. mackerrasae* were ATC(Ile) and ACC (Thr) and CTC (Leu), whereas it was TCC (Ser) for *A. vasorum*, TGC (Cys) for *A. cantonensis* and TGC (Cys), GAC (Asp), CTC (Leu) and ACC (Thr) for *A. costaricensis*. Of the 64 possible codons, 62 were used in mt genome of *A. mackerrasae*. Codons TCC (Ser) and CGC (Arg) were not used.

**Table 3:** Comparison of the positions of protein coding genes in the mt genomes of *Angiostrongylus mackerrasae, Angiostrongylus cantonensis, Angiostrongylus vasorum* and *Angiostrongylus costaricensis* and the start and stop codons for protein-coding genes as well as the lengths of their predicted amino acid sequences

Gene	Positions					Initiation/Termination codons and amino acid sequence lengths (aa)		
	*A. mackerrasae*	*A. vasorum*	*A. cantonensis*	*A. costaricensis*	*A. mackerrasae*	*A. vasorum*	*A. cantonensis*	*A. costaricensis*
*cox*1	1-1578	1 – 1573	1-1579	1-1579	ATT-TAA (525)	ATA-TAA (523)	ATT-TAG (525)	ATT-TAA (525)
*trn*C	1578-1637	1577 – 1634	1578-1634	1579-1634				
*trn*M	1637-1697	1637 – 1695	1637-1693	1635-1693				
*trn*D	1702-1755	1699 – 1755	1702-1754	1695-1748				
*trn*G	1755-1811	1755 – 1808	1755-1811	1752-1809				
*cox*2	1811-2503	1808 – 2504	1812-2505	1810-2503	TTG-TAG (230)	ATT-TAG (231)	TTG-TAG (230)	TTG-TAA (230)
*trn*H	2502-2557	2505 – 2560	2503-2557	2509-2564				
*rrn*L	2625-3586	2557 – 3518	2558-3519	2565-3531				
*nad*3	3586-3921	3521 – 3854	3517-3853	3531-3867	TTG-TAA (111)	TTG-TAG (111)	TTG-TAG (111)	TTG-TAG (111)
*nad*5	3924-5501	3886 – 5453	3855-5437	3880-5461	ATA-T (514)	ATA-T (544)	ATA-T (526)	ATA-T (526)
*trn*A	5506-5560	5453 – 5507	5437-5491	5462-5516				
*trn*P	5796-5850	5726 – 5783	5723-5777	5782-5835				
*trn*V	5856-5910	5787 – 5840	5782-5835	5838-5892				
*nad*6	5919-6344	5850 – 6276	5845-6271	5901-6333	ATG-TAG (141)	ATG-TAG (141)	ATG-TAG (141)	ATG-TAG (143)
*nad*4L	6345-6578	6279 – 6511	6271-6503	6333-6564	ATT-T (77)	ATT-T (76)	ATT-T (76)	ATT-T (76)
*trn*W	6577-6633	6511 – 6568	6503-6559	6565-6623				
*trn*E	6635-6691	6569 – 6623	6561-6615	6631-6689				
*rrn*S	6694-7390	6622 – 7318	6616-7312	6690-7385				
*trn*S (UCN)	7390-7444	7319 – 7375	7311-7366	7385-7439				
*trn*N	7445-7501	7374 – 7428	7365-7420	7438-7497				
*trn*Y	7506-7566	7433 – 7487	7425-7484	7502-7556				
*nad*1	7652-8500	7485 – 8361	7485-8361	7557-8430	ATT-TAG (291)	TTG-TAG (291)	TTG-TAG (292)	TTG-TAG (290)
*atp*6	8503-9102	8366 – 8963	8363-8963	8445-9045		ATT-TAA (198)	ATT-TAG (200)	ATT-TAG (200)
*trn*K	9104-9165	8966 – 9026	8964-9024	9046-9105				
*trn*L (UUR)	9165-9221	9029 – 9083	9025-9080	9107-9162				
*trn*S (AGN)	9268-10119	9084 – 9136	9080-9132	9163-9215				
*nad*2	9165-9221	9136 – 9979	9131-9980	9215-10064	TTG-TAG (283)	TTG-TAG (281)	TTG-TAG (282)	TTG-TAA (282)
*trn*I	10133-10189	9988 – 10045	9991-10047	10071-10124				
*trn*R	10189-10243	10045 – 10097	10048-10102	10125-10176				
*trn*Q	10244-10300	10098 – 10151	10102-10157	10178-10232				
*trn*F	10300-10356	10155 – 10211	10158-10213	10235-10291				
*cob*	10356-11465	10212 – 11319	10214-11324	10300-11401	TTG-TAA (369)	TTG-TAG (369)	TTG-TAA (369)	ATG-TAG (366)
*trn*L (CUN)	11465-11520	11326 – 11385	11323-11378	11401-11456				
*cox*3	11521-12291	11377 – 12145	11379-12145	11457-12225	TTG-TAG (256)	ATT-TAA (256)	TTG-T (255)	TTG-T (254)
*trn*T	12287-12344	12143 – 12199	12145-12202	12223-12280				
*nad*4	12345-13574	12199 – 13420	12203-13433	12281-13511	TTG-TAG (409)	TTG-TAG (406)	TTG-TAG (409)	TTG-TAA (409)

**Table 4:** Number of codons and percentage of codon usage (%) of the protein coding genes in mt genome of *A. mackerrasae*

		*Angiostrongylus mackerrasae*	*Angiostrongylus vasorum*	*Angiostrongylus cantonensis*	*Angiostrongylus costaricensis*
Non-polar					
Alanine	GCN	78(0.32)	88 (2.54)	75 (1.66)	52 (1.16)
Isoleucine	ATY	190 (5.52)	226 (6.53)	290 (6.40)	306 (6.80)
Leucine	CTN	25 (0.73)	23 (0.67)	135 (2.98)	152 (3.38)
Leucine	TTR	571 (16.59)	566 (16.36)	511 (11.28)	453 (10.07)
Methionine	ATR	212 (6.16)	148 (4.27)	225 (4.97)	191 (4.25)
Phenylalanine	TTY	472 (13.71)	461 (13.32)	614 (13.56)	675 (15.00)
Proline	CCN	77 (2.24)	71 (2.05)	57 (1.26)	35 (0.78)
Tryptophan	TGR	67 (1.95)	58 (1.68)	181 (4.00)	216 (4.80)
Valine	GTN	315 (9.15)	368 (10.63)	370 (8.17)	409 (9.09)
Polar					
Aspargine	AAY	115 (3.34)	92 (2.66)	146 (3.22)	155 (3.45)
Cysteine	TGY	51 (1.48)	77 (2.22)	156 (3.45)	209 (4.65)
Glutamine	CAR	41 (1.19)	38 (1.1)	46 (1.02)	32 (0.71)
Glycine	GGN	213 (6.19)	224 (6.47)	246 (5.43)	237 (5.27)
Serine	AGN	237 (6.89)	245 (7.08)	238 (5.29)	297 (6.56)
Serine	TCN	146 (4.24)	136 (3.94)	111 (2.48)	111 (2.45)
Threonine	ACN	88 (2.56)	77 (2.22)	102 (2.25)	56 (1.24)
Tyrosine	TAY	195 (5.66)	192 (5.55)	288 (6.36)	241 (5.36)
Acidic					
Aspartate	GAY	66 (1.92)	70 (2.02)	122 (2.69)	116 (2.58)
Glutamate	GAR	85(2.47)	80 (2.31)	105 (2.32)	131 (2.91)
Basic					
Arginine	CGN	32 (0.93)	161 (4.65)	33 (0.73)	34 (0.76)
Histidine	CAY	55 (1.60)	53 (1.53)	48 (1.06)	36 (0.80)
Lysine	AAR	101 (2.93)	93 (2.69)	161 (3.56)	155 (3.45)

### Transfer RNA and Ribosomal RNA genes

Twenty two tRNA genes were located in the mt genome of *A. mackerrasae*. The gene sequences ranged between 52 to 61 nt in length, identical to *A. vasorum* [11].

The *rrn*S and *rrn*L genes of *A. mackerrasae* were determined by sequence comparison with *A. cantonensis*, *A. costaricensis* and *A. vasorum*. As previously described for *A. vasorum* [11], the two genes were separated from each other by protein-encoding genes, including *nad*3, *nad*5 and *nad*4L (Fig. 1). The size of *rrn*S gene of *A. mackerrasae* was 696 bp and the *rrn*L was 961 bp. The size of both genes was identical to those described for *A. vasorum* [11] and *Bunostomum trigonocephalum* [16] and very similar to the size of rRNAs described previously for other nematodes (Table 5).

**Table 5:** The length of ribosomal RNA genes of *A. mackerrasae* in comparison with rRNA of other nematodes described previously

Species	*rrn*S	*rrn*L	Reference
*Angiostrongylus mackerrasae*	696	961	This study
*Angiostrongylus vasorum*	696	961	[11]
*Ascaris suum*	700	960	[11]
*Bunostomum trigonocephalum*	696	961	[15]
*Bunostomum phlebotomum*	694	961	[15]
*Caenorhabditis elegans*	697	953	[11]
*Onchocerca volvulus*	684	987	[28]
*Setaria digitata*	672	971	[28]
*Trichinella spiralis*	688	9047	[29]
*Trichuris discolor*	663	988	[30]
*Trichuris ovis*	699	989	[30]

### Genetic comparison between *A. mackerrasae *and other *Angiostrongylus *species, as well as other strongylid nematodes

**Fig. 2:**
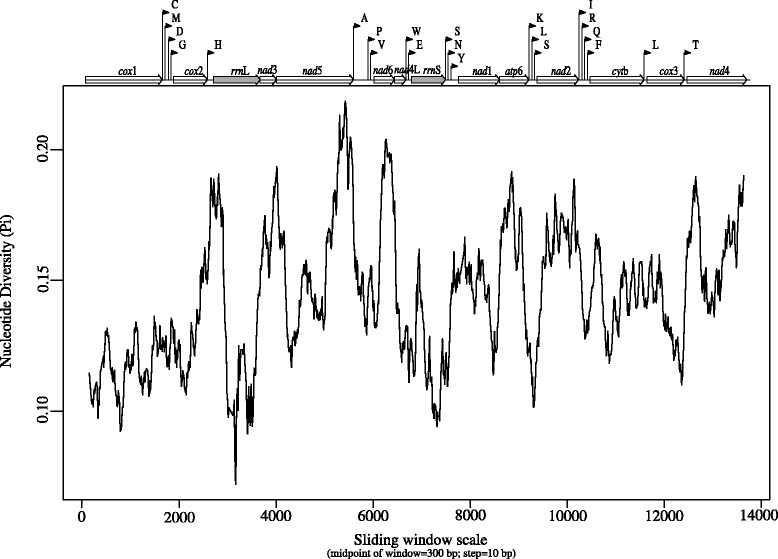
Sliding window analysis of complete mitochondrial genome of *Angiostrongylus mackerrasae.* The black line indicates nucleotide diversity in a window size of 300 bp

The analysis of nucleotide variation across the mt genomes between or among *A. mackerrasae*, *A. vasorum*, *A. cantonensis* and *A. costaricensis* showed the most diversity in the *rrn*L, *nad*6 and *atp*6 genes and in the 5’-end of *nad*5 and 5’- and 3’-ends of *nad*4. Least diversity was observed in the *cox*1, *cox*2 and *rrn*S genes (Fig. 2).

Pairwise comparisons of the concatenated amino acid sequences of *A. mackerrasae, A. cantonensis*, *A. costaricensis* and *A. vasorum* ranged from 70.63 to 99.57 % between different protein coding genes and showed higher identity between *A. mackerrasae* and *A. cantonensis*, ranging between 92.9 % (*nad4*L) and 99.57 % (*cox*2) (an average of 2.4 % difference between the two). The sequence identity revealed that *cox*1 was the most conserved protein among the four, while *nad*2, *nad*3 and *nad*6 were the least conserved proteins (Table 6). In addition, pairwise comparison of amino acid sequences among closely related species of strongylid nematodes showed that *A. mackerrasae* and *A. cantonensis* are the most closely related to congeners, followed by *Oesophagostomum quadrispinulatum* and *O. dentatum* (3.2 % difference) as well as *Ancylostoma caninum* and *A. duodenale* (4.0 % difference).

**Table 6:** Pairwise comparison (%) of the amino acid sequence predicted from each of the 12 protein coding mitochondrial genes from *Angiostrongylus mackerrasae*, *Angiostrongylus vasorum, Angiostrongylus cantonensis and Angiostrongylus costaricensis*

Predicted protein	*A. mackerrasae* vs *A. cantonensis*	*A. mackerrasae* vs *A. costaricensis*	*A. mackerrasae* vs *A. vasorum*
ATP6	97.49	77.39	84.92
COB	98.65	85.29	84.86
COX1	98.67	93.52	93.7
COX2	99.57	86.96	83.48
COX3	95.69	89.02	80.78
NAD1	92.91	81.91	84.04
NAD2	96.47	73.85	70.67
NAD3	97.3	72.97	77.48
NAD4	97.8	79.95	82.11
NAD4L	92.21	80.52	72.73
NAD5	97.67	78.6	75.1
NAD6	97.17	73.05	78.72

Using mt datasets, based upon pairwise comparisons of concatenated amino acid sequences predicted herein, we found considerable variation in the magnitude of sequence differences between closely related species of trichostrongyloids (14.9 % between *Trichostrongylus axei* and *T. vitrinus*); (19.9 % between *Haemonchus contortus* and *Mecistocirrus digitatus*), ancylostomatoids (4.0 % between *A. caninum* and *A. duodenale*); (11.4 % between *B. phlebotomum* and *B. trigonocephalum*), strongyloids (3.2 % between *Oe. dentatum* and *Oe. Quadrispinulatum*) and selected metastrongyloids (19.2 % between *D. eckerti* and *D. viviparus*; 13.6 % between *M. pudendotectus* and *M. salmi*; 16.8 % between *A. costaricensis* and *A.cantonensis* and 18.7 % between *A. costaricensis* and *A. vasorum*) (Table 7).

**Table 7:** Pairwise comparisons (sequence differences in %) among closely related species of strongylid nematodes

Species	Am	Aca	Aco	Av	De	Dv	Mp	Ms	Ta	Tv	Hc	Md	Ac	Ad	Bp	Bt	Od
Am																	
Aca	2.4																
Aco	16.8	16.8															
Av	16.9	16.9	18.7														
De	37.8	37.8	38.0	38.5													
Dv	36.8	36.9	36.9	38.2	19.2												
Mp	27.3	27.4	28.0	29.7	37.6	36.5											
Ms	26.5	26.8	27.2	29.2	37.6	36.9	13.6										
Ta	29.3	29.5	29.6	30.9	38.9	38.1	32.1	31.4									
Tv	30.0	30.0	29.9	31.8	38.3	37.9	32.6	31.9	14.9								
Hc	29.9	30.2	30.4	32.2	39.2	38.8	33.3	32.7	22.6	22.8							
Md	29.5	29.9	30.5	31.8	39.7	38.5	32.8	32.9	23.2	23.3	19.9						
Ac	27.4	27.6	28.3	29.9	37.7	37.2	30.9	30.6	21.4	20.1	22.1	22.2					
Ad	27.5	27.6	28.2	30.0	37.9	37.5	30.9	30.9	21.5	20.4	22.2	22.6	4.0				
Bp	27.1	27.3	27.6	29.8	37.7	36.5	30.5	30.2	22.0	21.6	23.8	23.4	14.8	15.0			
Bt	27.0	27.2	27.9	29.7	38.0	37.1	30.6	30.0	21.9	21.2	22.9	22.9	12.9	13.4	11.4		
Od	27.3	27.4	28.2	30.1	38.1	37.5	30.6	30.5	21.8	20.5	22.4	22.6	8.9	9.4	14.3	12.7	
Oq	27.4	27.6	28.5	30.4	38.2	37.6	30.9	30.9	21.8	20.6	22.5	22.7	9.3	10.2	14.5	13.0	3.2

*Am Angiostrongylus mackerrasae* (Strongylida: Angiostrongylidae), *Aca Angiostrongylus cantonensis* (Strongylida: Angiostrongylidae), Aco  *Angiostrongylus costaricensis* (Strongylida: Angiostrongylidae), *Av Angiostrongylus vasorum* (Strongylida: Angiostrongylidae), *De Dictyocaulus eckerti* (Strongylida: Dictyocaulidae), *Dv Dictyocaulus viviparus* (Strongylida: Dictyocaulidae), *Mp Metastrongylus pudendotectus* (Strongylida: Metastrongylidae), *Ms Metastrongylus salmi* (Strongylida: Metastrongylidae), *Ta Trichostrongylus axei* (Strongylida: Trichostrongylidae), *Tv Trichostrongylus vitrinus* (Strongylida: Trichostrongylidae), *Hc Haemonchus contortus* (Strongylida: Haemonchidae), *Md Mecistocirrus digitatus* (Strongylida: Haemonchidae), *Ac Ancylostoma caninum* (Strongylida: Ancylostomatidae), *Ad Ancylostoma duodenale* (Strongylida: Ancylostomatidae), *Bp Bunostomum phlebotomum* (Strongylida: Ancylostomatidae), *Bt Bunostomum trigonocephalum* (Strongylida: Ancylostomatidae), *Od Oesophagostomum dentatum* (Strongylida: Chabertiidae), *Oq Oesophagostomum quadrispinulatum* (Strongylida: Chabertiidae)

**Fig. 3:**
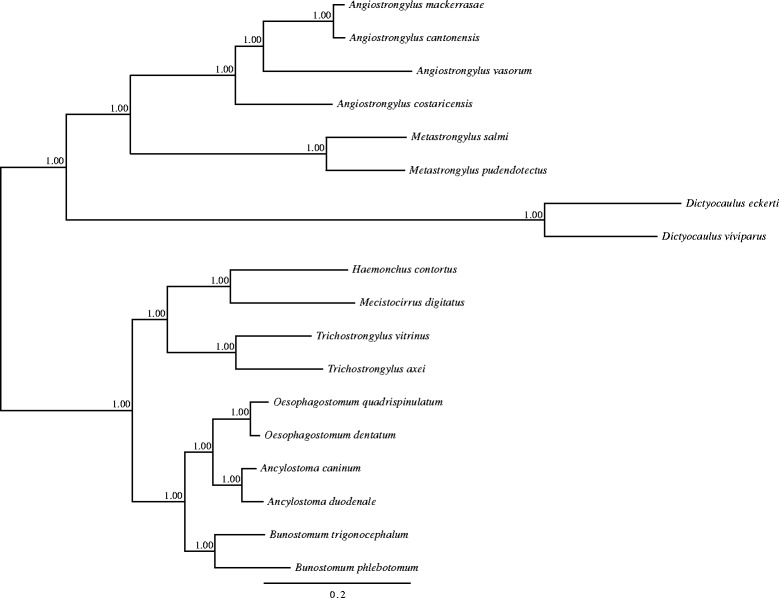
Relationship of *Angiostrongylus mackerrasae* with strongylid nematodes based on a phylogenetic analysis of concatenated amino acid sequence data for the 12 inferred mt proteins. There was absolute support (pp = 1.00) at each individual node

Extending these comparisons, phylogenetic analysis of the amino acid sequences encoded by the 12 mt genes revealed that *A. cantonensis* is the most closely related to *A. mackerrasae* among the four *Angiostrongylus* species. (Posterior probability (pp) =1:00). *Metastrongylus* spp. (Metastrongylidae) clustered separately from *Angiostrongylus* species but was the most closely related to the four *Angiostrongylus* species (pp = 1:00). Other strongylids such as *Dictyocaulus* spp. (Dictyocaulidae), *Trichostrongylus* spp. (Trichostrongylidae), *Haemonchus contortus* (Haemonchidae), *Mecistocirrus digitatus* (Haemonchidae); *Oesophagostomum* spp. (Chabertiidae); *Ancylostoma* spp. (Ancylostomatidae) and *Bunostomum* spp. (Ancylostomatidae) clustered separately from metastrongyloids (Fig. 3).

## Discussion

Mitochondrial sequences have been used as genetic markers for identification of organisms and interrelationships among diverse taxa [10, 17, 18]. Although nucleotide variation within species of nematodes is relatively high for the mt genes studied [18] and, thus, is not useful for specific identification, this is not the case for the inferred sequences of mt proteins. Amino acid sequence variation within species of nematodes is usually very low (0–1.3 %) [10, 18, 19]. Therefore, amino acid sequences inferred from the mt genomes provide species identification for studying the systematics (taxonomy and phylogeny) of nematodes [10]. Indeed, phylogenetic analysis of mt amino acid datasets usually provides strong statistical support for the relationships of nematodes, which is not achieved using data from short sequence tracts.

The amino acid sequence difference of 2.4 % across the entire predicted mt protein repertoire between *A. mackerrasae* and *A. cantonensis* is higher than the upper level of within-species sequence variation estimated to date (1.3 %), and similar to the lowest levels of sequence difference (3.2–4.0 %) between pairs of other closely related strongylid nematodes (i.e. *A. caninum* and *A. duodenale*; *Oe. dentatum* and *Oe. quadrispinulatum*) [18], providing support for the hypothesis that *A. mackerrasae* and *A. cantonensis* are separate species. Experimental hybridization by Bhaibulaya [20] has been shown to produce fertile female but sterile males (F1s), which provides biological evidence to support this proposal.

Similar specific distinctions on genetic grounds have been made for pairs of morphologically similar or identical species of strongylid that show distinct host preferences. Jabbar et al. [21] studied the mt genome of the strongyloid *Hypodontus macropi* from three different hosts species and concluded that the parasites from the different hosts represent three distinct species of *Hypodontus*. The lowest sequence difference between two *H. macropi* isolates from *Macropus robustus robustus* and *Macropus bicolour* was 5.8 %. Nonetheless, further study using independent, informative nuclear genetic markers is required to lend additional independent support for the two closely related *Angiostrongylus* species.

Given the close genetic identity but biological differences between the two species of Australian angiostrongylids, the origins and divergence of *A. mackerrasae* and *A. cantonensis* are interesting questions. It has been suggested that feral rats (species of *Rattus rattus* and *Rattus norvegicus*) were introduced to Australia with the first European ships in late 1700s [7]. Presumably, *A. cantonensis* arrived in Australia on ships travelling from Asia [22]. Considering the ongoing geographical expansion of *A. cantonensis* in Australia, based on the recent reports of parasite from human, dogs, rats and molluscs from NSW [23–26]*,* a phylogeographical analysis of this species is needed to resolve the question of origin of Australian populations of this species.

*Angiostrongylus mackerrasae* appears to be mainly specific to native *Rattus* (*R. fuscipes* and *R. lutreolus*) [3]*.* The native rat, *R. fuscipes,* is one of a number of Australian species that has been traced back to an invasion event in the final stages of the Pleistocene, when the Australian land mass was linked to Papua New Guinea (PNG) [7]. Molecular analysis of *Rattus* species in Australia demonstrates strong support for the specific identity of *R. fuscipes*, indicating that this species has not crossed with other Australian *Rattus* species, whereas the genetic fidelity of other *Rattus* species in Australia is less certain [27].

Did *A. mackerrasae* arise in Australia through natural invasions of a shared ancestor of the two parasite species from PNG? or did the current populations diverge from *A. cantonensis* populations after more recent introduction with European settlement and feral rat invasion? Bhaibulaya [20] favoured the more ancient, and northern, invasion by *A. mackerrasae*, explaining the morphologic similarity between the two species by hybridization and species introgression in the wild [20]. A challenge to this hypothesis is the apparent absence of any species of *Angiostrongylus* in tropical northern Australia. Dunsmore investigated rats on the Gulf of Carpentaria in the Northern Territory, but did not observe angiostrongylids [28] and there are no reports of eosinophilic meningitis in humans or animals from tropical Queensland, Australia. It should be noted however, that Dunsmore did not examine species of *Rattus* in his survey and may have missed evidence of the parasites. A recent survey of *Rattus* spp. in northern Queensland was also unable to show the presence of the parasite in tropical Queensland [29].

Anecdotal evidence from rodent trappers suggests that *R. fuscipes* actively excludes feral rats (*Rattus rattus* and *Rattus norvegicus*) from its habitats. Coupled with this, is the finding by Stokes et al. [23] that populations of *A. cantonensis* and *A. mackerrasae* were found in rats in different zones of forests of Jervis Bay, NSW. The evidence thus tentatively leans towards the view that despite their close genetic identity, the populations of *A. cantonensis* and *A. mackerrasae* are populations recently introduced into Australia. Accidental infection and establishment of populations in *R. fuscipes* has led to the two populations becoming isolated in terms of geographic habitat and host selection.

The occurrence of *A. mackerrasae* in Australia indicates a need to develop a molecular tool for the accurate/specific diagnosis of neural angiostrongyliasis in humans. Although *A. mackerrasae* has not been detected in humans, it has recently been recovered from a flying fox (*Pteropus alecto*) [5]. This raises questions as to the ability of *A. mackerrasae* to infect and cause disease in non-permissive hosts. There is even a possibility that *A. mackerrasae* is responsible for a portion of *Angiostrongylus* infections in humans in Australia. Yet, the focus of most studies of *Angiostrongylus* has been on *A. cantonensis* as it occurs in feral rats which live close to human dwellings. However, the expansion and encroachment of residential areas in Australia on forests has resulted in the native rats (e.g. *Rattus fuscipes*) being found in relatively close proximity to human habitation, potentially implicating *A. mackerrasae* as a potential zoonosis in these peri-urban regions. Moreover, current immunological [30] and molecular-based tools [31] for the detection of larvae in tissue target only *A. cantonensis.* If there is considerable divergence in protein sequence and immunological profiles of the two species, tools for diagnosis of neural angiostrongyliasis may not detect cases caused by *A. mackerrasae*.

The complete mt genome described here, now provides enough information to develop highly specific PCR-based tests to screen archival tissues of humans and dogs diagnosed with eosinophilic meningitis in order to distinguish the species of *Angiostrongylus* responsible for the infection. Genes such as *nad*4L showed a higher diversity between *A. cantonensis* and *A. mackerrasae* and could be a good region to be used in order to distinguish the two species.

The outcome of sliding window analysis in this study, offers valuable information of the high and low variability regions within the inter-species mt genome, providing useful data for population genetic studies and adds to the previously performed phylogenetic study of *Angiostrongylus* taxa by Eamsobhana et al. [32] which was restricted to the *cox*1 region of mt DNA and did not include *A. mackerrasae*.

## Conclusion

In conclusion, the present study emphasizes the importance and utility of the mt genomic datasets for nematodes from rodents, as a basis for the diagnosis of *A. mackerrasae* and *A. cantonensis* for ecological and biological studies of these nematodes. Importantly, the study also provides a stimulus to explore, in detail, the population genetics of these taxa across their distributional and host ranges using complete or partial (informative) mt genomic and protein sequence data sets. Although the present study focused on these two taxa, the approach used has important implications for investigating the systematics of a range of parasites (nematodes) from rodents, and defining genetic markers of utility to explore their epidemiology and population genetics. Future studies should focus on comparing multiple adult nematodes of *A. mackerrasae* and *A. cantonensis* from different geographical locations such as North and South eastern Australia and Southeast Asia, including PNG, to ascertain that the species sequenced in this study is not a hybrid.

## References

[1] Bhaibulaya M, Cross JH (1971). *Angiostrongylus malaysiensis* (Nematoda: Metastrongylidae), a new species of rat lung-worm from Malaysia. Southeast Asian J Trop Med Public Health.

[2] Kamis AB, Ahmad RA, Badrul-Munir MZ (1992). Worm burden and leukocyte response in *Angiostrongylus malaysiensis*-infected rats: the influence of testosterone. Parasitol Res.

[3] Spratt DM (2015). Species of *Angiostrongylus* (Nematoda: Metastrongyloidea) in wildlife: A review. Int J Parasitol Parasites Wildl.

[4] Bhaibulaya M (1968). A new species of *Angiostrongylus* in an Australian rat *Rattus fuscipes*. Parasitol.

[5] Mackie J, Lacasse C, Spratt D (2013). Patent *Angiostrongylus mackerrasae* infection in a black flying fox (*Pteropus alecto*). Aust Vet J.

[6] Aghazadeh M, Jones MK, Aland KV, Reid SA, Traub RJ, McCarthy JS, Lee R (2015). Emergence of neural angiostrongyliasis in eastern australia. Vector Borne Zoonotic Dis (Larchmont, NY).

[7] Robins J, McLenachan P, Phillips M, McComish B, Matisoo-Smith E, Ross H (2010). Evolutionary relationships and divergence times among the native rats of Australia. BMC Evol Biol.

[8] Stanisic J, Shea M, Potter D, Griffiths O. Australian Land Snails Volume 1: A Field Guide to Eastern Australian Species. Queensland Museum. Riviere des Anguilles, Mauritius: Bioculture Press; 2010.

[9] Hu M, Jex AR, Campbell BE, Gasser RB (2007). Long PCR amplification of the entire mitochondrial genome from individual helminths for direct sequencing. Nat Protoc.

[10] Jex AR, Hall RS, Littlewood DT, Gasser RB (2010). An integrated pipeline for next-generation sequencing and annotation of mitochondrial genomes. Nucleic Acids Res.

[11] Gasser RB, Jabbar A, Mohandas N, Schnyder M, Deplazes P, Littlewood DT, Jex AR (2012). Mitochondrial genome of *Angiostrongylus vasorum*: comparison with congeners and implications for studying the population genetics and epidemiology of this parasite. Infect Genet Evol.

[12] Lv S, Zhang Y, Zhang L, Liu Q, Liu HX, Hu L, Wei FR, Steinmann P, Graeff-Teixeira C, Zhou XN (2012). The complete mitochondrial genome of the rodent intra-arterial nematodes *Angiostrongylus cantonensis* and *Angiostrongylus costaricensis*. Parasitol Res.

[13] Edgar RC (2004). MUSCLE: multiple sequence alignment with high accuracy and high throughput. Nucleic Acids Res.

[14] Huelsenbeck JP, Ronquist F (2001). MRBAYES: Bayesian inference of phylogenetic trees. Bioinformatics.

[15] Jabbar A, Mohandas N, Jex AR, Gasser RB (2013). The mitochondrial genome of *Protostrongylus rufescens* - implications for population and systematic studies. Parasit Vectors.

[16] Gao JF, Zhao Q, Liu GH, Zhang Y, Zhang Y, Wang WT, Chang QC, Wang CR, Zhu XQ (2014). Comparative analyses of the complete mitochondrial genomes of the two ruminant hookworms *Bunostomum trigonocephalum* and *Bunostomum phlebotomum*. Gene.

[17] Le TH, Blair D, McManus DP (2002). Mitochondrial genomes of parasitic flatworms. Trends Parasitol.

[18] Hu M, Gasser RB (2006). Mitochondrial genomes of parasitic nematodes--progress and perspectives. Trends Parasitol.

[19] Hu M, Chilton NB, Gasser RB (2004). The mitochondrial genomics of parasitic nematodes of socio-economic importance: recent progress, and implications for population genetics and systematics. Advances Parasitol.

[20] Bhaibulaya M (1974). Experimental hybridization of *Angiostrongylus mackerrasae*, Bhaibulaya, 1968 and *Angiostrongylus cantonensis* (Chen, 1935). Int J Parasitol.

[21] Jabbar A, Beveridge I, Mohandas N, Chilton NB, Littlewood DT, Jex AR, Gasser RB (2013). Analyses of mitochondrial amino acid sequence datasets support the proposal that specimens of *Hypodontus macropi* from three species of macropodid hosts represent distinct species. BMC Evol Biol.

[22] Banks PB, Hughes NK. A review of the evidence for potential impacts of black rats (*Rattus rattus*) on wildlife and humans in Australia. Wildlife Res. 2012;39.

[23] Stokes VL, Spratt DM, Banks PB, Pechc RP, Williams RL (2007). Occurrence of Angiostrongylus species (Nematoda) in populations of *Rattus rattus* and *Rattus fuscipes* in coastal forests of south-eastern Australia. Aust J Zoo.

[24] Morton NJ, Britton P, Palasanthiran P, Bye A, Sugo E, Kesson A, Ardern-Holmes S, Snelling TL (2013). Severe Hemorrhagic Meningoencephalitis Due to *Angiostrongylus cantonensis* Among Young Children in Sydney, Australia. Clin Infect Dis.

[25] Chan D, Barratt J, Roberts T, Lee R, Shea M, Marriott D, Harkness J, Malik R, Jones M, Aghazadeh M (2015). The Prevalence of *Angiostrongylus cantonensis*/*mackerrasae* Complex in Molluscs from the Sydney Region. PloS one.

[26] Walker AG, Spielman D, Malik R, Graham K, Ralph E, Linton M, Ward MP (2015). Canine neural angiostrongylosis: a case–control study in Sydney dogs. Aust Vet J.

[27] Robins JH, Tintinger V, Aplin KP, Hingston M, Matisoo-Smith E, Penny D, Lavery SD (2014). Phylogenetic species identification in *Rattus* highlights rapid radiation and morphological similarity of New Guinean species. PloS one.

[28] Dunsmore JD (1968). Absence of *Angiostrongylus cantonensis* from the Gulf of Carpentaria in Northern Australia. J Parasitol.

[29] Aghazadeh M, Reid SA, Aland KV, Restrepo AC, Traub RJ, McCarthy JS, Jones MK (2015). A survey of *Angiostrongylus* species in definitive hosts in Queensland. Int J Parasitol Parasites Wildl.

[30] Maleewong W, Sombatsawat P, Intapan PM, Wongkham C, Chotmongkol V (2001). Immunoblot evaluation of the specificity of the 29-kDa antigen from young adult female worms *Angiostrongylus cantonensis* for immunodiagnosis of human angiostrongyliasis. Asian Pac J Allergy Immunol.

[31] Qvarnstrom Y, da Silva ACA, Teem JL, Hollingsworth R, Bishop H, Graeff-Teixeira C, da Silva AJ (2010). Improved Molecular Detection of *Angiostrongylus cantonensis* in Mollusks and Other Environmental Samples with a Species-Specific Internal Transcribed Spacer 1-Based TaqMan Assay. Appl Environ Microbiol.

[32] Eamsobhana P, Lim PE, Zhang H, Gan X, Yong HS (2010). Molecular differentiation and phylogenetic relationships of three Angiostrongylus species and *Angiostrongylus cantonensis* geographical isolates based on a 66-kDa protein gene of *A. cantonensis* (Nematoda: Angiostrongylidae). Exp Parasitol.

